# Antifungal resistance in patients with Candidaemia: a retrospective cohort study

**DOI:** 10.1186/s12879-019-4710-z

**Published:** 2020-01-17

**Authors:** Namareq F. Aldardeer, Hadiel Albar, Majda Al-Attas, Abdelmoneim Eldali, Mohammed Qutub, Ashraf Hassanien, Basem Alraddadi

**Affiliations:** 1 King Faisal Specialist Hospital and Research Center, Jeddah, Saudi Arabia; 2 King Faisal Specialist Hospital and Research Center, Riyadh, Saudi Arabia; 3 Anti-infective, Pfizer, Jeddah, Saudi Arabia; 4 Alfaisal University, Riyadh, Saudi Arabia

**Keywords:** Antifungal resistance, Epidemiology, Risk factors, *Candida parapsilosis*, Mortality, Fluconazole resistance

## Abstract

**Background:**

Candidaemia is the most common form of invasive candidiasis. Resistant *Candida* blood stream infection (BSI) is rising, with limitations on the development of broader-spectrum antifungal agents worldwide. Our study aimed to identify the occurrence of antifungal-resistant candidaemia and the distribution of these species, determine the risk factors associated with antifungal resistance and evaluate the association of antifungal-resistant candidaemia with the length of intensive care unit (ICU) and hospital stay and with 30-day mortality.

**Methods:**

A retrospective cohort study was conducted at King Faisal Specialist Hospital and Research Centre, Jeddah, Saudi Arabia. Adult patients diagnosed with candidaemia from January 2006 to December 2017 were included.

**Results:**

A total of 196 BSIs were identified in 94 males (49.74%) and 95 females (50.26%). *C. glabrata* was the most commonly isolated *Candida* species, with 59 (30%), followed by *C. albicans* with 46 (23%). Susceptibility data were available for 122/189 patients, of whom 26/122 (21%) were resistant to one or more antifungals. *C. parapsilosis* with available sensitivity data were found in 30/122 isolates, of which 10/30 (33%) were resistant to fluconazole. Risk factors significantly associated with antifungal-resistant candidaemia included previous echinocandin exposure (odds ratio (OR) =1.38; 95% confidence interval (CI) (1.02–1.85); *P* = 0.006) and invasive ventilation (OR = 1.3; 95% CI (1.08–1.57); *P* = 0.005). The median length of ICU stay was 29 days [range 12–49 days] in the antifungal-resistant group and 18 days [range 6.7–37.5 days] in the antifungal-sensitive group (*P* = 0.28). The median length of hospital stay was 51 days [range 21–138 days] in the antifungal-resistant group and 35 days [range 17–77 days] in the antifungal-sensitive group (*P* = 0.09). Thirty-day mortality was 15 (57.7%) and 54 (56.25%) among the antifungal-resistant and antifungal-sensitive groups, respectively (OR = 1.01; 95% CI (0.84–1.21); *P* = 0.89).

**Conclusions:**

Our results indicate a high frequancy of non- *C. albicans candidaemia*. The rise in *C. parapsilosis* resistance to fluconazole is alarming. Further studies are required to confirm this finding.

## Background

Systemic fungal infections have emerged as a significant public health problem [1]. Among hospitalized patients, candidaemia is the most common form of invasive candidiasis (IC), which accounts for 9% of all nosocomial bloodstream infections (BSIs) [2, 3]. In a 2014 United States (US) surveillance study, *Candida* species ranked as the seventh causative organism of healthcare-associated BSIs [4]. Recent reports indicated that candidaemia is the third or fourth most common hospital-acquired BSI in US hospitals [5]. *Candida* species are considered the leading pathogen in many fungal infections affecting humans. Among 15 distinct *Candida* species causing human disease, *C. albicans, C. glabrata, C. tropicalis, C. parapsilosis* and *C. krusei* are the most common pathogens leading to IC [5].

Risk factors associated with antifungal resistance have been addressed in many studies. Previous fluconazole exposure, neutropenia and chronic kidney disease are known to contribute to *C. albicans* fluconazole resistance [6]. However, total parental nutrition, previous episodes of candidaemia and the presence of fluconazole-resistant isolates were mainly accompanied by echinocandin resistance [7]. In Saudi Arabia, many studies have described the distribution and risk factors for *Candida* BSI; however, there are no local studies on the risk factors associated with antifungal resistance and its effect on mortality. *C. albicans* were the most commonly isolated *Candida* among all studies conducted in Saudi Arabia [8–11]. Predisposing factors related to candidaemia occurrence were related to use of a central venous catheter, previous use of broad-spectrum antibiotics and complicated abdominal surgeries; the mortality rate was 43% for all candidaemia cases [9]. Therefore, our study was conducted to identify the occurrence of antifungal-resistant candidaemia, describe the distribution of *Candida* species among hospitalized patients with candidaemia, determine the risk factors associated with antifungal resistance and evaluate the association of antifungal resistance with length of ICU and hospital stay and with 30-day mortality.

## Methods

### Setting and study design

A retrospective cohort study was conducted at King Faisal Specialist Hospital and Research Centre, Jeddah, Saudi Arabia (KFSH&RC-J), a 500-bed teaching and tertiary care hospital. The study was approved by the hospital institutional review board. Between January 2006 and December 2017, all adult patients 18 years old and above with isolated *Candida* BSI with or without other forms of IC were included. IC included disseminated hepatosplenic candidiasis, intra-abdominal candidiasis, *Candida* intravascular infection, osteoarticular candidiasis, *Candida* endophthalmitis, vulvovaginal candidiasis, *Candida* urinary tract infection, oropharyngeal candidiasis, oesophageal candidiasis or central nervous system candidiasis [5]. The study excluded patients under 18 years of age and those infected with IC alone in the absence of candidaemia. Patients with candidaemia were reviewed through hospital electronic charts. The REDCap system was used to collect patient information, such as patient demographics and comorbidities. Baseline characteristics and *Candida* distribution were assessed for all patients with *Candida* BSI. Patients with available susceptibility data were divided into two groups: antifungal-sensitive and antifungal-resistant; risk factor and mortality analyses were performed. Antifungal resistance was considered for any identified isolate with resistance to one or more antifungals based on the laboratory cut-off value of minimum inhibitory concentration (MIC).

The primary outcome was the occurrence of antifungal resistance among candidaemia patients over a 12-year study period. The secondary outcomes were the distribution of *Candida* BSI, the risk factors for antifungal resistance and the association of antifungal resistance with length of ICU and hospital stay and with 30-day mortality.

### Microbiology

The identification of *Candida* BSI was consistent throughout the study period. Identification was performed by a blood culture test and processed by using the automated blood culture system BACTEC FX. The average incubation period was 5 days and reaches up to 21 days whenever candidaemia is suspected. The API 20C AUX Kit was used for identification and specification of yeast, and Sensititre YeastOne was used for susceptibility. The MICs for fluconazole, voriconazole, itraconazole, posaconazole, amphotericin B, flucytosine, caspofungin, anidulafungin and micafungin were interpreted based on the Clinical and Laboratory Standard Institute (CLSI M27-S4). As the interpretation of the amphotericin B susceptibility breakpoint has not yet been defined [12], our study did not address amphotericin B susceptibility testing for *Candida* BSI.

### Statistical analysis

Data were analysed using SAS version 9.4. Descriptive statistics using the mean, median and interquartile range (IQR) were used for continuous variables. Frequencies and percentages were used for categorical variables. The chi-square test and Fisher’s exact test were used to compare categorical variables. For risk factor analysis of antifungal resistance, we performed a univariate logistic regression analysis. For 30-day mortality, we performed univariate and multivariable analyses to identify factors associated with mortality. A *P* value less than 0.05 was considered significant.

## Results

From January 2006 to December 2017, a total of 196 *Candida* species were identified in 189 patients. Demographic characteristics and risk factors associated with resistant *Candida* BSI are listed in (Table 1). The most common *Candida* species during the study period was *C. glabrata* (*n* = 59, 30.1%). Distributions of *Candida* species are included in (Table 2). Table.

**Table 1:** Baseline characteristics for patients with candida bloodstream infection (BSIs)

Characteristics	Number of Patients (*N* = 189) n (%)
Mean Age (years)	57.3 ± 17.5
Gender	
Male	94 (49.74%)
Female	95 (50.26%)
Bacteremia co-infection	79 (41.8%)
Baseline Comorbidities	
Diabetes	99 (52.38%)
Hypertension	105 (55.56%)
Chronic kidney disease	24 (12.7%)
Liver Cirrhosis	33 (17.46%)
Cardiovascular diseases	96 (50.79%)
Lung diseases	52 (27.51%)
Human immunodeficiency virus	3 (1.59%)
Solid organ transplantation	8 (4.23%)
Hematological malignancy	9 (4.76%)
Non hematological malignancy	48 (25.4%)
Neutropenia ^a^	25 (13.23%)
History of IV drug user	3 (1.59%)
Blood/ Platelet transfusion	135 (71.43%)
Recent hospital admission	35 (18.52%)
Exposure to medication within 3 months ^a^	
Broad spectrum antibiotics	182 (96.3%)
Polyene antifungal	3(1.59%)
Azole antifungal (Fluconazole)	11 (5.82%)
Echinocandins	38 (20.11%)
Corticosteroids	86 (45.5%)
Exposure to medical procedures within 3 months	
TPN	20 (10.58%)
Central line insertion	151 (79.89%)
Urine catheter	148 (78.31%)
Invasive Ventilation	100 (52.91%)
Surgery within 3 months	77 (40.74%)
GI	38 (49.35%)
Non- GI	39 (50.65%)
Renal Replacement therapy	66 (34.92%)
HD	54 (28.57%)
PD	4 (2.12%)
CRRT	11 (5.82%)
SLID	4 (2.12%)

Total parental nutrition (TPN); Gastrointestinal (GI); Hemodialysis (HD); Peritoneal dialysis (PD); Continuous renal replacement therapy (CRRT); Sustained low efficiency dialysis (SLID); Intravenous (IV);^a^Neutropenia defined as an absolute neutrophil count of, 1.0 10^9^/L or less; N = total number of patients; n = number of patients or events

**Table 2:** Distribution of *Candida* Species over the study period

*Candida* isolates	Total number of isolates (*N* = 196) n(%)
*C. glabrata*	59 (30.1)
*C. albicans*	46 (23.47)
*C. parapsilosis*	38 (19.39)
*C. tropicalis*	35 (17.86)
*C. krusei*	6 (3.06)
*C. famata*	5 (2.55)
*C. lusitaniae*	1 (0.51)
*C .kefyr*	1 (0.51)
*C. dubliniensis*	1 (0.51)
*C. trichosporon asahii*	1 (0.51)
Others	3 (1.53)

One hundred and twenty-two patients with antifungal susceptibility data were analysed in the candidaemia resistance risk factor analysis (Table 3); the percentage of antifungal resistance was 21% (26/122). Candidaemia with antifungal resistance was significantly more likely to develop among patients with previous echinocandin exposure (odds ratio (OR), 1.38; 95% CI (1.02–1.85); *P* = 0.006) and in patients who received invasive ventilation (OR, 1.3; 95% CI (1.08–1.57); *P* = 0.005).

**Table 3:** Univariate regression analysis for risk factors associated with antifungal resistance candidemia

	Antifungal sensitive (*N* = 96)	Antifungal resistance (*N* = 26)	OR (95% CI)	*P* value
Age (years) Mean ± SD	58.7 ± 16.2	56.5 ± 20.29		
Gender, n (%)				
Male	53 (55.21)	10 (38.46)	0.5 (0.2_1.23)	0.12
Female	43 (44.79)	16 (61.54)		
Comorbidities, n (%)				
Diabetes	61 (63.54%)	15 (57.69%)	0.94 (0.77_1.15)	0.58
Hypertension	56 (58.33%)	16 (61.54%)	1.02 (0.85_1.23)	0.76
Chronic Kidney disease	14 (14.58%)	4 (15.38%)	1.01 (0.77_1.32)	0.91
Liver Cirrhosis	10 (10.42%)	4 (15.38%)	1.11(0.78_1.57)	0.48
Cardiovascular disease	44 (45.83)	16 (61.54)	1.14(0.94_1.37)	0.15
Lung diseases	24 (25%)	6 (23.08%)	0.97 (0.79_1.2)	0.83
Solid organ transplantation	2 (2.08%)	2 (7.69%)	1.59 (0.59_4.26)	0.15
Hematological malignancy	2 (2.08%)	0		
Non Hematological malignancy	35 (36.46%)	2 (7.69%)	0.75 (0.65_0.88)	0.004
Neutropenia	7 (7.29%)	4 (15.38%)	1.09 (0.31_3.79)	0.88
History of IV drug user	2 (2.08%)	1 (3.85%)	1.18 (0.52_2.65)	0.6
3-months exposure before candidemia				
Blood/ Platelet transfusion	60 (62.5%)	20 (76.92%)	1.14 (0.95_1.36)	0.16
Recent hospital admission	20 (20.83%)	8 (30.77%)	1.13 (0.87_1.45)	0.28
Broad spectrum antibiotics	90 (93.75%)	26 (100%)	1.28 (1.16_1.42)	0.19
Polyene antifungal	3 (3.13)	0	0.78 (0.71_0.85)	0.36
Azole antifungal (fluconazole)	8 (8.33%)	1 (3.85%)	0.87 (0.68_1.12)	0.43
Echinocandins	19 (19.79%)	12 (46.15%)	1.38 (1.02_1.85)	0.006
Corticosteroids	52 (54.17%)	19 (73.08%)	1.17 (0.98_1.4)	0.08
TPN	13 (13.54%)	4 (15.38%)	1.03 (0.78_1.36)	0.81
Central catheter insertion	71 (73.96%)	21 (80.77%)	1.07 (0.88_1.31)	0.47
Urine catheter	73 (76.04%)	17 (65.38%)	0.88 (0.69_1.12)	0.27
Invasive Ventilation	44 (45.83%)	20 (76.92%)	1.3 (1.08_1.57)	0.005
Previous surgery	38 (39.58%)	14 (53.85%)	1.13 (0.93_1.37)	0.19
GI	21(55.26%)	4 (28.5%)	1.08 (0.88_1.31)	0.42
Non- GI	17 (44.7%)	10 (71.4%)		
Renal Replacement therapy	36 (37.5%)	12 (46.15%)	1.08 (0.88_1.31)	0.42
HD	26 (27.08)	12 (46.15)	1.21 (0.96_1.54)	0.06
PD	3 (3.13)	0		
CRRT	9 (9.38)	0		
SLID	2 (2.08)	2 (7.69)	1.59 (0.59–4.2)	0.15

Odd ratio (OR); Confidence interval (CI); Total parental nutrition (TPN); Gastrointestinal (GI); Hemodialysis (HD); Peritoneal dialysis (PD); Continuous renal replacement therapy (CRRT); Sustained low efficiency dialysis (SLID) N = total number of patients; n = number of patients or events

Cross-resistance among azole antifungals was observed in our study. All strains resistant to voriconazole, including five strains of *C. albicans*, two strains of *C. parapsilosis* and one strain of *C. tropicalis,* were also resistant to fluconazole. Only two isolates were resistant to caspofungin, and three isolates were resistant to anidulafungin (Additional file 1: Tables S1 and S2).

The median (interquartile range [IQR]) length of ICU stay was 29 days [range 12–49 days] in the antifungal-resistant group and 18 days [range 6.7–37.5 days] in the antifungal-sensitive group (*P* = 0.28). The median [IQR] length of hospital stay was 51 days [range 21–138 days] in the antifungal-resistant group and 35 days [range 17–77 days] in the antifungal-sensitive group (*P* = 0.09). Thirty-day mortality was 15 (57.7%) and 54 (56.25%) for the antifungal-resistant and antifungal-sensitive groups, respectively [OR = 1.01; 95% CI (0.84–1.21); *P* = 0.89] (Table 4).

**Table 4:** Outcome analysis for patients with antifungal resistance candidemia

	Antifungal sensitive (*N* = 96)	One or more antifungal resistance (*N* = 26)	*P* Value
Length of ICU Stay (*n* = 84), Median (IQR)	18 (6.7 _ 37.5)	29 (12 _ 49)	0.28
Length of hospital stay (days) Median (IQR)	35 (17_ 77)	51.5 (21_ 138)	0.09
Mortality before first negative culture n (%)	26 (27.08)	5 (19.23)	0.41
30-day mortality n (%)	54 (56.25)	15 (57.69)	0.89

Interquartile range (IQR), N = total number of patients; n = number of patients or events

In a multivariate logistic regression analysis used to assess factors associated with mortality in patients with candidaemia, the following factors were independently associated with mortality. Liver cirrhosis [OR = 5.36; 95% CI (1.14–25.1)], non-haematological malignancy [OR = 2.3; 95% CI (1.01–5.25)], blood/platelet transfusion [OR = 4.4; 95% CI (1.98–9.7)], central venous catheter [OR = 4.37; 95% CI (1.79–10.66)] and invasive ventilation [OR = 3.88; 95% CI (1.82–8.29)] (Additional file 1: Table S3).

We performed a secondary analysis for patients with fluconazole-resistant *C. parapsilosis* to identify risk factors for resistance. Univariate analysis revealed that echinocandin exposure [OR = 12.8; 95% CI (2.01–81.10)], blood/platelet transfusion [OR = 14.6; 95% CI (1.54–138.18)] and invasive ventilation [OR = 2.1; 95% CI (1.31–3.39)] were associated with fluconazole-resistant *C. parapsilosis* (Table 5).

**Table 5:** Univariate regression analysis for statistically significant risk factors associated with fluconazole resistance C.*parapsilosis* in patients with candidemia

	Fluconazole sensitive *C.parapsilosis* (*N* = 20)	Fluconazole resistance *C.parapsilosis* (*N* = 10)	OR (95%CI)	*P* Value
Previous Echinocandin Exposure	5 (23.81%)	8 (80%)	12.8 (2.01_81.1)	0.005
Blood and Platelet Transfusion	7 (35%)	9 (90%)	14.6 (1.54_138.18)	0.006
Invasive Ventilation	9 (42.8%)	10 (100%)	2.1 (1.31_3.39)	0.004

Odd ratio (OR), N = total number of patients

## Discussion

**Fig. 1:**
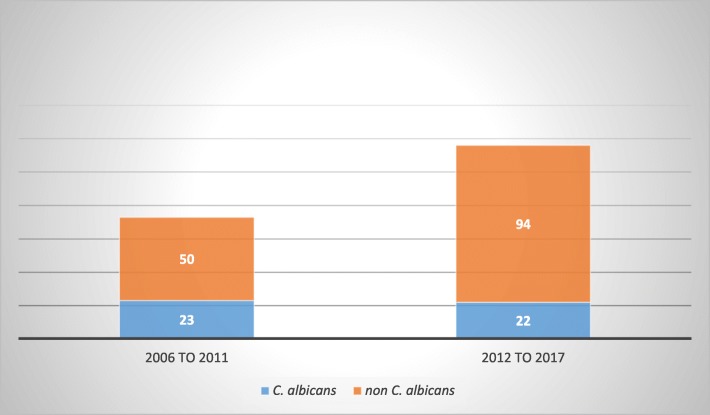
Distribution of *Candida* Species over the study period

Worldwide, there has been a notable shift away from *C. albicans* among patients with *Candida* BSI [13]. Our study showed a high frequency in non-*C. albicans* candidaemia (Fig. 1). These strains had a higher cost and length of hospital stay than *C. albicans* [14]. *C. glabrata* was the predominantly isolated *Candida* species among our candidaemia patients, representing 30 % of cases, followed by *C. albicans* and *C. parapsilosis*. These findings differ from those of previous local studies. Two studies conducted in Saudi Arabia in a university hospital and armed force hospital between 1998 to 1999 and 1996 to 2002 concluded that *C. albicans* was the most commonly isolated *Candida* species among patients with candidaemia, followed by *C. tropicalis* and *C. parapsilosis* [8, 11]. Moreover, *C. albicans* remained the dominant isolated species among candidaemia cases in many studies [1, 2, 15, 16]. Geographic variation significantly affects the species distribution of *Candida* [2]. A systematic review summarizing the distribution of *Candida* species found a high concentration of *C. albicans* isolates in Northern and Central Europe in addition to the USA; however, non*-C. albicans* species were more common in South America, Asia and Southern Europe [17].

Antifungal resistance is a growing primary concern for most healthcare providers. We analysed one hundred and twenty-two *Candida* BSI cases for resistance. Twenty-one percent of all isolates were resistant to one or more antifungal agents. The patient’s risk of developing antifungal resistance was a primary concern in our study. We looked for many possible predisposing factors for drug insensitivity 90 days prior to a candidaemia episode. Patient’s previous exposures to broad-spectrum antibiotics, antifungals and corticosteroids were reported; none of the aforementioned risk factors showed a significant association with antifungal resistance in our study despite a positive association of previous antimicrobial exposure to fluconazole resistance among candidaemia in a previous study [18]. However, previous exposure to echinocandins was significantly associated with antifungal resistance among our patients. Many researchers have confirmed that decreased susceptibility to *Candida* is significantly associated with previous antifungal exposure and an inappropriate prior course of antifungal therapy [6, 19, 20]. In fact, fluconazole exposure was found to be a risk factor for gene mutation and overexposure that leads to future fluconazole-resistant *C. parapsilosis* [21]. Furthermore, we investigated patient comorbidities and history of invasive procedures or surgery. Many studies addressed drug-resistant candidaemia among cancer patients and considered them a high-risk group [22]. However, our cohort found that antifungal resistance was low among candidaemia patients with non-haematological malignancy. On the other hand, invasive ventilation was significantly associated with drug resistance; this result has been concluded by many researchers, who found that *Candida* resistance is dramatically higher among critical-care patients [17].

*C. parapsilosis* is known to have a high affinity for fluconazole, and resistance was absent in an earlier prospective trial [23]. In our study, up to 33 % of *C. parapsilosis* strains were insensitive to fluconazole. Clinical resistance to echinocandins is rare; however, some cases of caspofungin resistance in patients with prolonged exposure to echinocandins have been reported [13]. Our study found few cases of echinocandin resistance. A 2012 US study investigated changes in the incidence of antifungal drug resistance, showing a 7% resistance to fluconazole and only 1% to echinocandin [15]. In a US multicentre candidaemia surveillance programme, an increase in the rate of echinocandin resistance was mainly found in *C. glabrata*, despite it being the preferred treatment [7]. Indeed, patients with fluconazole resistance were at higher risk for *C. glabrata*-associated echinocandin resistance [16]. The variation in antifungal resistance patterns across geographic regions was addressed in a report on SENTRY antimicrobial surveillance, which observed a detectable resistance to anidulafungin, micafungin, and azoles among isolates of *C. glabrata* from North America [24].

*Candida* BSI contributed to a prolonged hospital stay and an increase in the overall healthcare cost [2]. Furthermore, drug resistance was associated with overall mortality [10, 22]. Despite the increase in length of hospital stay and mortality among our patients with antifungal resistance, the results were not significant.

The retrospective nature of our study could prevent us from identifying all risk factors related to our candidaemia cases. Data on source control and appropriateness of antifungal treatment in terms of timing and dosing of antifungals were not included in our study. Patients who transferred from an outside hospital had no previous records in our system, and risk factors were identified only by physician evaluation notes and nurse documentation. Another limitation is the unavailability of susceptibility data between 2006 and 2011 for all *Candida* species isolated during our study period, as the request for data was sent out and performed only upon physician request. Although KFSHRC-J is a bone marrow transplantation centre, the number of patients with haematological malignancies involved in our study was small, which prevents us from investigating fluconazole pre-exposure as a risk factor for drug-resistant candidaemia.

## Conclusions

The study identified a high frequency of non-*albicans* candidaemia. The rise in fluconazole-resistant *C. parapsilosis* is alarming. More regional data from different hospital settings are needed to allow for a comparison of findings. Our study emphasizes the importance of implementation and compliance with antimicrobial stewardship programmes to control antifungal utilization among hospitalized patients.

## Supplementary information

**Additional file 1: Table S1.** Resistance pattern among candidemia patients with available susceptibility data by *Candida* isolates. **Table S2.** Resistance pattern among candidemia patients with available susceptibility data by antifungal group. **Table S3.** Multivariate regression analysis for the statistically significant risk factors affecting mortality in patients with antifungal resistance candidemia.

## Data Availability

The datasets used and/or analysed during the current study are available from the corresponding author on reasonable request.

## Abbreviations

BSI: Bloodstream Infection
CI: Confidence Interval
CLSI: Clinical and Laboratory Standard Institute
CRRT: Continuous Renal Replacement Therapy
GI: Gastrointestinal
HD: Haemodialysis
IC: Invasive Candidiasis
ICU: Intensive Care Unit
IQR: Interquartile Range
KFSH&RC-J: King Faisal Specialist Hospital and Research Centre, Jeddah
MIC: Minimum Inhibitory Concentration
OR: Odds Ratio
PD: Peritoneal Dialysis
TPN: Total Parental Nutrition
US: United States
