# Supplementary material for: Antifungal resistance in patients with Candidaemia: a retrospective cohort study
Source: BMC Infect Dis. 2020 Jan 17;20:55. doi: 10.1186/s12879-019-4710-z (PMC6969401; doi:10.1186/s12879-019-4710-z)
Supplement: Supplementary file 1 — Additional file 1: Table S1. Resistance pattern among candidemia patients with available susceptibility data by Candida isolates. Table S2. Resistance pattern among candidemia patients with available susceptibility data by antifungal group. Table S3. Multivariate regression analysis for the statistically significant risk factors affecting mortality in patients with antifungal resistance candidemia. [file 12879_2019_4710_MOESM1_ESM.docx]

**Supplementary Appendix:**

| **Table S1: Resistance Pattern among Candidemia Patients with available Susceptibility Data by *Candida* isolates** | | | | | |
| --- | --- | --- | --- | --- | --- |
| ***Candida* isolates with available susceptibility data** | **Resistant to Fluconazole** | **Resistant to Voriconazole** | **Resistant to Anidulafungin** | **Resistant to Caspofungin** | **Resistant to at least one antifungal** |
| ***C. glabrata (n=43)*** | 3 | 0 | 0 | 0 | 3 (6.9%) |
| ***C. parapsilosis (n=30)*** | 10 | 2 | 1 | 1 | 14 (46.7%) |
| ***C. albicans (n=26)*** | 4 | 4 | 0 | 0 | 8 (30.7%) |
| ***C. tropicalis (n=15)*** | 1 | 1 | 2 | 1 | 5 (33.3%) |
| ***C. krusei (n=5)*** | 5 | 0 | 0 | 0 | 5 (100%) |
| ***Others (n=3)*** | 0 | 0 | 0 | 0 | - |
| ***Total isolates (n =122)*** | 23 (18.8%) | 7 (5.7%) | 3 (2.4%) | 2 (1.6%) | 35 (28.8%) |

| **Table S2: Resistance Pattern among Candidemia Patients with available Susceptibility Data by Antifungal group** | | | |
| --- | --- | --- | --- |
| ***Candida* isolates with available susceptibility data** | **Resistant to Azoles*** | **Resistant to Echinocandins**** | **Resistant to at least one antifungal** |
| ***C. glabrata (n=43)*** | 3 | 0 |  |
| ***C. parapsilosis (n=30)*** | 10 | 1 |  |
| ***C. albicans (n=26)*** | 4 | 0 |  |
| ***C. tropicalis (n=15)*** | 1 | 2 |  |
| ***C. krusei (n=5)*** | 5 | 0 |  |
| ***Others (n=3)*** | 0 | 0 |  |
| ***Total isolates (n =122)*** | 23 (18.8%) | 3 (2.4%) | 26 (21.3%) |
| * patients who were resistant to voriconazole and fluconazole were calculated once as azole resistance  ** patients who were resistant to capsofungin and anidulafungin were calculated once as echinocandins resistance | | | |

| **Table S3: Multivariate Regression analysis for the statistically significant risk factors affecting mortality in patients with antifungal resistance candidemia** | | |
| --- | --- | --- |
|  | **OR (95% CI)** | **P value** |
| **Age** | 0.97 (0.95_0.99) | 0.016 |
| **Not Resistant** | 0.94 (0.39_2.26) | 0.89 |
| **Liver Cirrhosis** | 5.36 (1.14_25.1) | 0.02 |
| **Non-hematological Malignancy** | 2.3 (1.01_5.25) | 0.049 |
| **Blood and Platelet Transfusion** | 4.4 (1.98_9.7) | 0.002 |
| **Central line** | 4.37 (1.79_10.66) | 0.001 |
| **Invasive Ventilation** | 3.88 (1.82_8.29) | 0.0005 |
| Odd ratio (**OR**) | | |
